# Supplementary material for: Thirst interneurons that promote water seeking and limit feeding behavior in Drosophila
Source: eLife. 2021 May 21;10:e66286. doi: 10.7554/eLife.66286 (PMC8139827; doi:10.7554/eLife.66286)
Supplement: Supplementary file 1. [file elife-66286-supp1.docx]

| **InSITE Strain** | **Seeking Index** |
| --- | --- |
| **530** | -0.331 |
| **600** | -0.313 |
| **507** | -0.286 |
| **1317** | -0.282 |
| **1276** | -0.252 |
| **1249** | -0.252 |
| **519** | -0.248 |
| **279** | -0.223 |
| **1296** | -0.174 |
| **1277** | -0.174 |
| **273** | -0.169 |
| **1247** | -0.167 |
| **189** | -0.145 |
| **1278** | -0.141 |
| **1293** | -0.138 |
| **1260** | -0.132 |
| **250** | -0.125 |
| **1299** | -0.124 |
| **1259** | -0.114 |
| **1312** | -0.110 |
| **1303** | -0.106 |
| **1318** | -0.105 |
| **602** | -0.104 |
| **1282** | -0.104 |
| **1258** | -0.102 |
| **604** | -0.097 |
| **588** | -0.093 |
| **1337** | -0.090 |
| **1339** | -0.090 |
| **1305** | -0.084 |
| **570** | -0.080 |
| **403** | -0.079 |
| **1334** | -0.078 |
| **584** | -0.076 |
| **1291** | -0.076 |
| **1308** | -0.075 |
| **372** | -0.072 |
| **1292** | -0.068 |
| **1251** | -0.062 |
| **1353** | -0.061 |
| **1269** | -0.059 |
| **1289** | -0.058 |
| **1236** | -0.057 |
| **1360** | -0.057 |
| **1328** | -0.053 |
| **1375** | -0.053 |
| **1233** | -0.050 |
| **1272** | -0.045 |
| **1244** | -0.045 |
| **1319** | -0.039 |
| **1300** | -0.038 |
| **1314** | -0.033 |
| **1264** | -0.033 |
| **1320** | -0.029 |
| **172** | -0.027 |
| **1302** | -0.026 |
| **107** | -0.025 |
| **1301** | -0.024 |
| **1271** | -0.023 |
| **1298** | -0.021 |
| **1287** | -0.021 |
| **1288** | -0.020 |
| **1270** | -0.019 |
| **1306** | -0.019 |
| **176** | -0.017 |
| **1330** | -0.017 |
| **1327** | -0.017 |
| **1273** | -0.014 |
| **1231** | -0.014 |
| **1332** | -0.014 |
| **1323** | -0.007 |
| **279** | -0.006 |
| **1338** | -0.005 |
| **1252** | 0.000 |
| **28** | 0.001 |
| **80** | 0.002 |
| **520** | 0.003 |
| **1325** | 0.005 |
| **1274** | 0.006 |
| **1286** | 0.006 |
| **277** | 0.007 |
| **46** | 0.010 |
| **1250** | 0.010 |
| **1261** | 0.014 |
| **492** | 0.019 |
| **1336** | 0.020 |
| **177** | 0.026 |
| **1313** | 0.035 |
| **1247** | 0.035 |
| **1256** | 0.035 |
| **1329** | 0.036 |
| **320** | 0.037 |
| **1316** | 0.038 |
| **1341** | 0.039 |
| **1239** | 0.039 |
| **1284** | 0.048 |
| **1253** | 0.049 |
| **1277** | 0.053 |
| **496** | 0.057 |
| **1267** | 0.057 |
| **340** | 0.060 |
| **1238** | 0.063 |
| **138** | 0.067 |
| **1342** | 0.070 |
| **1255** | 0.072 |
| **1294** | 0.075 |
| **1245** | 0.076 |
| **1335** | 0.078 |
| **533** | 0.084 |
| **1310** | 0.084 |
| **1242** | 0.092 |
| **557** | 0.095 |
| **102** | 0.097 |
| **158** | 0.099 |
| **192** | 0.101 |
| **1254** | 0.104 |
| **461** | 0.110 |
| **1265** | 0.128 |
| **1315** | 0.141 |
| **1263** | 0.142 |
| **1304** | 0.147 |
| **72** | 0.149 |
| **1237** | 0.155 |
| **1279** | 0.157 |
| **249** | 0.166 |
| **95** | 0.171 |
| **1321** | 0.171 |
| **208** | 0.173 |
| **1248** | 0.174 |
| **1240** | 0.190 |
| **21** | 0.191 |
| **441** | 0.196 |
| **349** | 0.209 |
| **1266** | 0.210 |
| **799** | 0.222 |
| **553** | 0.226 |
| **1379** | 0.226 |
| **1280** | 0.268 |
| **89** | 0.269 |
| **269** | 0.270 |
| **47** | 0.289 |
| **226** | 0.298 |
| **1264** | 0.314 |
| **260** | 0.365 |
| **560** | 0.378 |
| **382** | 0.389 |
| **501** | 0.425 |
| **9** | 0.484 |
| **1285** | 0.496 |
| **152** | 0.505 |
| **1234** | 0.688 |
